# Supplementary material for: QTL Analysis for Rice Quality-Related Traits and Fine Mapping of qWCR3
Source: Int J Mol Sci. 2024 Apr 16;25(8):4389. doi: 10.3390/ijms25084389 (PMC11050666; doi:10.3390/ijms25084389)
Supplement: Supplementary file 1 [file ijms-25-04389-s001.zip › ijms-2919972-supplementary.pdf]

Table S1 Primers used in this study.

Name	Forward primer (5'-3')	Reverse primer (3'-5')
2.32.3	ATCATGTGCTAATGGAATGC	GTCAATACCAGGAATCTCTTG
3.16.1	AACGCAAACAACAACACGAA	CCCCGAGATATCCATCAACA
4.30.6	GATGATACAGGGTGATTAGTG	AGATGATGCCAAATTAGGTG
4.34.0	TGCACTTAAGAAACGTGTAAGC	GTGCTTTGTCGGTGCCATAG
9.2.0	TCTGCTTTCCAACCGAAATC	GGTTTCTGGTGTTTGGCTTT
9.8.0	CAGCGTACACTAGGCACACG	TGTTTAAAAGTCAACAACGCACA
10.0.1	ACCTTTTGCTGTTCGCAGAT	TCAGGTGTGTGTACCTATGGTG
11.6.6	TTATGAGGATAACCATGCTTGC	TATGGGCTAAATTACACGCTTTG
A1.40.39	CAACAACGAGGACGATGA	CGAACAAACTGGCTAATGAA
A10.284	CATGACTCGTGAGTAGCA	CCAAACATTACCAAGTTCTG
AY4292	CCTTTGCAATGGTGAGCGAT	ATCAGCTCTAGCCATGCTCC
B1.40.61	CTTCACCTACCAACCAATTC	CAGCAGCAGTAACTGTAAC
B10.389	CCAGACACTGACAGTTAGG	ACATCATTTCACACGGTTG
C1.23.3	TGCGTGTGATTTCCCTTG	GTACTTATGCACCACCTGTA
C3.26.6	CACATGAAGCTCGACCAT	ACACATCGCTCAGAATTAAC
C9-12.3	CCTCACCATACTCGACATC	GGATGGACACCTTGATCG
C10.13.5	GAATAAGGACTTGGTGTTGAG	CGCATTTCACCCAAAGAAC
C10.17.4	CAACGAGAAGCTGCTTGC	GCATACACTACAGTGCTCAA
C11-3.5	GAAGCATTTGCCAAAGAAC	AGAAATACCGTCCAATCTCT

Name	Forward primer (5'-3')	Reverse primer (3'-5')
D1-8	TTGGGTGAAGATGCAATATAATAAA	TTTATCAACACCCAAGTACATCA
D2-1	GCAGATCAAGAGCTCAATGG	CAATCAACACTCACATTCTCAGC
D2-6	GCTGCATCGATGGGAATAAG	CGCCAACTTACAAGCGATGT
D3-6	ATAATACAGGGAGGAGCAAAGC	CGCCGCATCCAGATTTATTA
D5-5	CGAGTATGATGGCCTAGACTTGA	TTGATCAAACTCCTCAATTCCTC
D5-6	CGATCATTGTCCACTTGACC	CAGGCGAATTAACGTGATGA
D10-11	TGCTAATCCTTATATTCCCTCTGTG	TGCTGTCCAAAGGACTGAGA
D11-8	TGGAATAAAATTAAACCCTTGAGC	GCAGGAAAATTCAAGAGCAGA
DD10-2	GAGCTCACTATTCGCACTTCC	GCTAGAAACTTGTAATTTTTCTTTTGC
DD10-4	AGATGAGCTTGGTGCAACACT	CCAATTGCCGTTGTCCAT
H1.25.1	GCTCACTAAGATCCCATTGA	GACGGTAGTGTGATGAAGT
H9.4.7	GCTGAGTGCCACTGAAAT	GTAGACGACCGCTTGATG
RM5	TGCAACTTCTAGCTGCTCGA	GCATCCGATCTTGATGGG
RM11	TCTCCTCTTCCCCCGATC	ATAGCGGGCGAGGCTTAG
RM14	CCGAGGAGAGGAGTTCGAC	GTGCCAATTTCCTCGAAAAA
RM21	ACAGTATTCCGTAGGCACGG	GCTCCATGAGGGTGGTAGAG
RM31	GATCACGATCCACTGGAGCT	AAGTCCATTACTCTCCTCCC
RM104	GGAAGAGGAGAGAAAGATGTGTGTCG	TCAACAGACACACCGCCACCGC
RM140	TGCCTCTTCCCTGGCTCCCCTG	GGCATGCCGAATGAAATGCATG
RM160	AGCTAGCAGCTATAGCTTAGCTGGAGATCG	TCTCATCGCCATGCGAGGCCTC
RM162	GCCAGCAAAACCAGGGATCCGG	CAAGGTCTTGTGCGGCTTGCGG
RM164	TCTTGCCCGTCACTGCAGATATCC	GCAGCCCTAATGCTACAATTCTTC
RM172	TGCAGCTGCGCCACAGCCATAG	CAACCACGACACCGCCGTGTTG
RM209	ATATGAGTTGCTGTCGTGCG	CAACTTGCATCCTCCCCTCC
RM218	TGGTCAAACCAAGGTCCTTC	GACATACATTCTACCCCCGG

Name	Forward primer (5’-3’)	Reverse primer (3’-5’)
RM220	GGAAGGTAACTGTTTCCAAC	GAAATGCTTCCCACATGTCT
RM228	CTGGCCATTAGTCCTTGG	GCTTGCGGCTCTGCTTAC
RM259	TGGAGTTTGAGAGGAGGG	CTTGTTGCATGGTGCCATGT
RM264	GTTGCGTCCTACTGCTACTTC	GATCCGTGTCGATGATTAGC
RM267	TGCAGACATAGAGAAGGAAGTG	AGCAACAGCACAACTTGATG
RM297	TCTTTGGAGGCGAGCTGAG	CGAAGGGTACATCTGCTTAG
RM310	CCAAAACATTTAAAATATCATG	GCTTGTTGGTCATTACCATTC
RM327	CTACTCCTCTGTCCCTCCTCTC	CCAGCTAGACACAATCGAGC
RM336	CTTACAGAGAAACGGCATCG	GCTGGTTTGTTTCAGGTTCG
RM338	CACAGGAGCAGGAGAAGAGC	GGCAAACCGATCACTCAGTC
RM340	GGTAAATGGACAATCCTATGGC	GACAAATATAAGGGCAGTGTGC
RM345	ATTGGTAGCTCAATGCAAGC	GTGCAACAACCCCACATG
RM346	CGAGAGAGCCCATAACTACG	ACAAGACGACGAGGAGGGAC
RM404	CCAATCATTAACCCCTGAGC	GCCTTCATGCTTCAGAAGAC
RM405	TCACACACTGACAGTCTGAC	AATGTGGCACGTGAGGTAAG
RM406	GAGGGAGAAAGGTGGACATG	TGTGCTCCTTGGGAAGAAAG
RM407	GATTGAGGAGACGAGCCATC	CTTTTTCAGATCTGCGCTCC
RM409	CCGTCTCTTGCTAGGGATTC	GGGGTGTTTTGCTTTCTCTG
RM412	CACTTGAGAAAGTTAGTGCAGC	CCCAAACACACCCAAATAC
RM414	ATTGCAGTCATGCAGCAGTC	ATATCTCCAATGTGGCAGGG
RM418	TCGCGTATCGTCATGCATAG	GAGCACATATGCCACGTACG
RM420	GGACAGAATGTGAAGACAGTCG	ACTAATCCACCAACGCATCC
RM434	GCCTCATCCCTCTAACCCTC	CAAGAAAGATCAGTGCGTGG
RM439	CTGGGTCTAATCTCGTCCTAAATTGC	CGCCTCTCATAACAGTCCACTCC
RM459	CTGCAATGCTGCATGACC	CACTTTCTCTGCAGCACCAG

Name	Forward primer (5’-3’)	Reverse primer (3’-5’)
RM478	CAGCTGGGGAAGAGAGAGAG	TCAGAAACTAAACGCACCCC
RM480	TGGTACTCACCATGCAAGTAGAACG	ATGCTCAAGCATTCTGCAGTTGG
RM481	TAGCTAGCCGATTGAATGGC	CTCCACCTCCTATGTTGTTG
RM486	CCCCCCTCTCTCTCTCTCTC	TAGCCACATCAACAGCTTGC
RM488	AACAACCAGCGTATGCGTTCTCG	CCCACGGCTTTGTAGGAAGAAGC
RM501	GCCCAATTAATGTACAGGCG	ATATCGTTTAGCCGTGCTGC
RM509	TAGTGAGGGAGTGGAAACGG	ATCGTCCCCACAATCTCATC
RM515	TAGGACGACCAAAGGGTGAG	TGGCCTGCTCTCTCTCTCTC
RM518	CTCTTCACTCACTCACCATGG	ATCCATCTGGAGCAAGCAAC
RM525	GGCCCGTCCAAGAAATATTG	CGGTGAGACAGAATCCTTACG
RM527	GGCTCGATCTAGAAAATCCG	TTGCACAGGTTGCGATAGAG
RM536	TCTCTCCTCTTGTTTGGCTC	ACACACCAACACGACCACAC
RM541	TATAACCGACCTCAGTGCCC	CCTTACTCCCATGCCATGAG
RM545	CAATGGCAGAGACCCAAAAG	CTGGCATGTAACGACAGTGG
RM556	ACTCCAAACCTCACTGCACC	TAGCACACTGAACAGCTGGC
RM583	AGATCCATCCCTGTGGAGAG	GCGAACTCGCGTTGTAATC
RM586	ACCTCGCGTTATTAGGTACCC	GAGATACGCCAACGAGATACC
T2-1	GGAGATAAGAACTCCATCCACAA	GCTACGTTCATTTCGCCATT
T2-2	CCAACTGCCGATTTGTTCA	CGAATTGTTTTCTATGAAATTCCTG
T3-1	CATTGGTTGGTAATTGGATGG	TGTGCCACATCCACTCATTAT
T3-4	TACAAAGGTGCGCAGCATAC	CCTGCTGTGTGTGGGAAAG
T4-6	TCCGAGCTTGATGAGATTGA	GCTTTTTGGCAGTGCACATT
T7-3	AACCTAGAGGCCGGGAGTAT	CCGCATTCATTCATATAATTGTG
TT2-4	CAACGCCTACAGCAACAT	CGGATCTTGGGATGAGTTC
TT4-8	GTGACTGTGAGCAGATGA	GGAATCAGGATTACAGTAGATG

Name	Forward primer (5’-3’)	Reverse primer (3’-5’)
TT5-1	GGTAGATTCTGGTTGCCTATA	GCTAGTGGAGAAAGAACTAAAG
TT5-2	GGCGGTTAGATCATCAGG	CCTTCTCCACATCCAACTC
TT6-1	ACTGTTCGCTCGTAAGTG	GCTTAGCCACAAGTGAAAC
TT9-3	TATAATGGAAGGTCTGGAGAG	GAATCTATCGGTCGGGAAT
Z1-18.0	CCCATACACATATCTACATTCC	ATTGGTCAGCGTGAAAGT
Z1-28.1	GCTATAAGACACGGATGCT	GAATGACTGTGAGTTAAGGAG
Z2-23.9	TTGGATCACTAGCCTGCAAA	TGGATATCTCACGGCTTTGTC
Z2-25.7	GCACCTAGCAATATACACTAC	CAGACTACTCTTCCGATGG
Z3-10.3	TTTACGAGGTTGGGTTTCG	CCGGTTGATACATCACCCTAT
Z3-11.0	TTCCTATTCATTCAGCACGA	GCTCCTTTGGACCGTTAA
Z3-14.8	TGGTTGGTTAGTAGACTGAA	CCGTCCACCTCACATTGA
Z3-17.6	TATATCGCTGCCTGATGC	GCGGAAGCAGTAACCTTG
Z3-29.1	GTCCATTTGTTCACCTAAAGG	TTGGCAGCGGAAATCATT
Z4-4.0	GAAGGACATCAAGGAGATATG	CAGATCTTGCATAGAACGAA
Z4-6.0	CCAACAAAGAGGAACGCAAT	ATTCGCAAATTTGTCCATCA
Z4-12.0	ATGGTGGTGGTATGGAGG	GCTTCTGAAAGTACGTTTGG
Z4-14.3	TGCTTGTGTCCTATAGTGG	AATTCCTTGCAGTTCTTGTC
Z4-17.7	ATAGATGGTGCGGCAGTT	GCTTTACTCGCTTCTTCACA
Z4-20.6	GGGTTTGAATTCTAGAGACG	GCTTCCCACCTTCTCTTT
Z4-26.5	GAGAAGGCTCACTCTATCAT	ATGTAACTCAACGGATGGAT
Z4-35.1	GGATACCTTGGATTGGAGTG	CCCGTCTTTAATACTGTTTCC
Z5-5.1	AAGTGAGGCGTGAGTCTC	CTTCCACTGACATCCACATAA
Z5-7.2	GGTGTAAGTAGATGCAGCA	ATCCAATGATGCGTACTACT
Z5-9.7	GCTCTTCCTTAAAGGCAATG	AAGTCTCTGTAAACACTCCA
Z5-13.0	CCGACCATAGGATTAGACAA	ATCACCGCATCACCAGAT

Name	Forward primer (5’-3’)	Reverse primer (3’-5’)
Z5-24.3	TGGTCTCACTCTCTGTTAT	CCATCTACTGTTTACTCAAG
Z6-21.9	TCGTATTCACATCCGAGGAA	GCATGTAGCCGATGACCTTA
Z6-6.0	GATGGTATACTGTCCTTGTAAG	AACTGATAATTCACCCTTGG
Z7-3.9	GTGGTCCCTAACTCAAACA	GGAAACGTCACATTCATAGA
Z7-5.4	CAGTCAACCGACGAGATT	GTGTTTCTACCTTATCCTCCT
Z7-11.7	CGACGACACAACAAACAC	GGCATTCTTTCCTTTCAACA
Z7-24.5	TTAACTTGCTCTGACTGAGA	GCCTCCAGACTAGACCAA
Z7-28.3	CGGTGGTGCTCTATCAGT	TCGTTCACATCCAAACAAGT
Z7-29.1	CGCAATGTTCGATACTGAA	AACTGAGGCATTGGTAGTAA
Z8-8.1	TTGCATGAGGGCTGGTTTAT	GGCCCTCCGGAGATTTTA
Z8-17.5	CCGCAATCCGAAGACTCT	AGCATCTCAGAAACTGGTATG
Z8-19.4	CCTAATCACCGCCTTCCA	TGACATAGATGGTAGCATAGAC
Z8-25.3	GCATTGGCACACCTAGAC	CTGGAAAGTAAGTGCGATTT
Z9-3.4	TTGCTAGGAAGGTAACTGAC	GTCTGTACGCTCCACATG
Z9-10.4	GCGAATCATATGGCAACAC	CGGCGTGCTTGAAGAATT
Z9-21.4	TTCATTGCTACTCGAACGG	GCGTGAGCAATCTATTATCC
Z9-22.4	CCATTTACACACGTTCACAT	AGTAGGCTTCTCACATTTCA
Z10-8.0	AAAGGGAAAGGAAGAACCAA	TGGCTGGAATCTCGTACA
Z10-16.6	TGTAGTTTGCGAGGACAAG	TAGCCCAGTGTTCAATTCTT
Z11-16.9	ACAACATCTTGCCTAAGC	AAGCAGTATGAGGTTAGAGA
Z11-27.8	GACAAATCAATAGGGCAACA	GCACCGCCTTATTCAAAG
Z12-0.9	GTCTCAGAATTTGGCTTGTG	CCGCATCTTGTATTCTTACC
Z12-2.2	CGATGGTTGTCACAATCTATAC	CGTCTACTTGTGTGATGGTA
Z12-4.0	GCTGGTCTTATATTAAGTAGGG	TCACGAGATTATCTGCCTAG
Z12-5.2	ACCAAACTAAGGCAGCAC	TTAGGTCACGGAGAAGATTG

Name	Forward primer (5’-3’)	Reverse primer (3’-5’)
Z12-9.4	GTTACGGCATGGTCCTAT	GCATAGCTCTTCTCTTTAGAC
Z12-13.5	GACCCGCCTTATCCACTT	TTAACAAATGTCGCATCCTC
Z12-16.4	CGCCAAAGGTAATCAATCAT	GAAGTAGAACGGTAATGTGAG
Z12-18.1	GAAAGAGCCTGTTGTTAGTT	TGTAATTCACTGGGAGTACC
Z12-19.9	TTGCCGAAGAACAAATGTAC	AACCTTGTCACTGCATCTC
Z12-22.1	ATATCAAGAGCGATGTGGTA	CATAGTTCTGCAATGGTGTT
Z12-25.2	ACGCAAGAATGAGATGTTC	ACGTAGAGCTGGATAAAGTA
WBa2	AGCCTCTAGAAGAAATGTGTTGC	TATATGATGAGCCCCGCCAA
WBa3	TTTGTTACGCTTGGCCCATC	ACTCCACGTTAAACACCTTTTTAT
WBa7	AAAATCGTATCAAGATTGGCGC	GAGAGTGCGGCTCAATCG
WBa9	AGGAAGGAAGGAAAGGGGTG	GTGTCCAACCAATCAGCTGC
WBa2.8	CCAAAGGACACCATATCTGCA	CCGTGTGGTCTATGTGAACAG
WBa3.07	TTACGTCACGGCAAAGGGTA	ACACTCACATTCTCAGCTGC
W11-2.17	GACTGTGACAGTGGAGGGTT	ACCTTCCTCACCTTGGGATG
W11-8.65	TGGGGGATTGCTGATATGCA	ACAGTTGGGCATTTTAACCAGT
24.57	GGCTCCAGGGAAGGGAAG	ATCCAATCCAAAACCAGCCG
25.07	AGAGATGCAGTGCTGTATA	GAGAGGGTGTGATCCAAA
25.15	TTTGGATCTCAACACGCGAA	GAAATGGGTGAAGATAGTGAGCT
25.36	CATCTACACCACGCATAGT	TGCCCGATTAGTATAGGTATAG
25.49	AATTCAAGTGATGCGAGGTA	CCATGGCCGTGGATTCAAC
25.52	TGTCCTATCCGGTCCTAGGT	TGAGGTGCAACATAAGCTAAAAGA
25.59	TCAACAACACTCATCCACTT	GGAAACGAACAAGACCTATATG
25.83	GACCCCGGCGATACTAAAGT	ACCACATTGAAAATTTGAAGGGA
26.21	CTCTCCTCTCGCTCAACTCC	CTGGTTAACCAAGCAACCGT
26.44	TCCACCTAACTCTACTCTCA	CCTGACTGCTAGTACCATAA
